# Quantitative assessment of muscle injury by ^23^Na magnetic resonance imaging

**DOI:** 10.1186/s40064-016-2193-6

**Published:** 2016-05-23

**Authors:** Anke Dahlmann, Christoph Kopp, Peter Linz, Alexander Cavallaro, Hannes Seuss, Kai-Uwe Eckardt, Friedrich C. Luft, Jens Titze, Michael Uder, Matthias Hammon

**Affiliations:** Department of Nephrology and Hypertension, Friedrich-Alexander-University Erlangen-Nuremberg, Erlangen, Germany; Department of Radiology, University Hospital Erlangen, Friedrich-Alexander-University Erlangen-Nuremberg, Maximiliansplatz 1, 91054 Erlangen, Germany; Experimental and Clinical Research Centre, A joint cooperation between the Charité Medical Faculty, The Max-Delbrück Centre for Molecular Medicine (MDC), Berlin, Germany; Department of Clinical Pharmacology, Vanderbilt University, Nashville, TN USA

**Keywords:** Magnetic resonance imaging, Sodium, Sports injury, Healing, Assessment, Quantification

## Abstract

**Background:**

^23^Na magnetic resonance imaging (^23^Na-MRI) is able to measure Na^+^ in vivo in humans and allows quantification of tissue sodium distribution. We now tested the utility of ^23^Na-MRI technique in detecting and assessing sports-related acute muscular injury.

**Case presentation:**

We assessed tissue Na^+^ of both lower legs with a 3T MRI scanner using a customized ^23^Na knee coil. The affected left calf muscle in an injured volleyball player showed a hyperintense Na^+^ signal. Follow-up measurements revealed persistently increased muscle Na^+^ content despite complete clinical recovery.

**Conclusions:**

Our findings suggest that ^23^Na-MRI could have utility in detecting subtle muscular injury and might indicate when complete healing has occurred. Furthermore, ^23^Na-MRI suggests the presence of substantial injury-related muscle electrolyte shifts that warrant more detailed investigation.

## Background

^23^Na magnetic resonance imaging (MRI) is a novel technique that allows in vivo quantification of tissue Na^+^ distribution. We developed this tool to investigate primary and secondary hypertension, changes in body and serum Na^+^ concentrations and Na^+^ shifts in patients undergoing dialysis (Kopp et al. 2013, 2012a, b). Others have developed similar techniques to inspect Na^+^ abnormalities in skeletal muscle diseases (Lehmann-Horn et al. 2012; Weber et al. 2011). Sports-related trauma leads to acute muscle injury that is oftentimes not easy to assess clinically. Conventional ^1^H-MRI can be helpful in detecting edema and structural changes. We were interested whether or not ^23^Na-MRI could also have utility in detecting and quantitatively assessing sports-related injury.

## Case presentation

A 35 year-old woman presented with acute sharp pain in her left calf. The pain suddenly appeared during a volley ball game. The patient could not recall any trauma. There was pain with walking and on the next day she noted swelling of the affected area. The physical examination was otherwise entirely normal. Arterial blood supply and venous drainage of the left lower leg were unremarkable. There was no indication of fracture and neurologically, the extremity was intact. We suspected torn fibers in the *triceps surae* and prescribed cooling and elevation of the injured lower leg.

We performed ^23^Na- and ^1^H-MR imaging with a 3 Tesla scanner (Magnetom Trio, Siemens Healthcare GmbH, Erlangen, Germany) of both lower legs. We used a customized ^23^Na knee coil as described previously (Kopp et al. 2013, 2012a, b; Hammon et al. 2015a, b). A gradient echo ^23^Na sequence was applied (total acquisition time TA: 3.25 min, echo time TE: 2.07 ms, repetition time TR: 100 ms, flip angle FA: 90°, 32 averages, resolution: 3 × 3 × 30 mm^3^). We additionally performed a T1-weighted fast-low-angle-shot (FLASH)-sequence for anatomic information. The scanning protocol is shown in Table 1. To calibrate Na^+^ signals, calibration tubes with 10, 20, 30 and 40 mmol/l NaCl were arranged below both calf muscles. Gray-scale measurements of the tubes served as calibration standards for ^23^Na-MRI by relating intensity to a concentration in a linear trend analysis. We calibrated these techniques in earlier studies. Amputated lower limbs from subjects undergoing operations because of malignancy or diabetes were measured with ^23^Na-MRI. These limbs were desiccated (the difference between wet weight and dry weight was considered tissue water content) and ashed and measured with atomic absorption spectrometry, allowing us to show a very close correlation between ^23^Na-MRI signal and actual Na^+^ concentrations in muscle and skin (Kopp et al. 2013; Dahlmann et al. 2015).

**Table 1:** Scanning protocol

	Localizer	T1-weighted fast-low-angle-shot (FLASH)-sequence	Gradient echo ^23^Na sequence (acquired 4 times successively)
Total acquisition time (TA; min)	0.15	2.08	3.25
Echo time (TE; ms)	4	2.46	2.07
Repetition time (TR; ms)	8.6	250	100
Flip angle (FA; °)	20	60	90
Averages	2	2	32
Bandwidth (Hz/pixel)	320	310	430
Field of view (FoV; mm)	192	192	192
Matrix (pixel)	256	256	64
Resolution (mm)	0.75 × 0.75 × 10	0.75 × 0.75 × 5	3 × 3 × 30

**Fig. 1:**
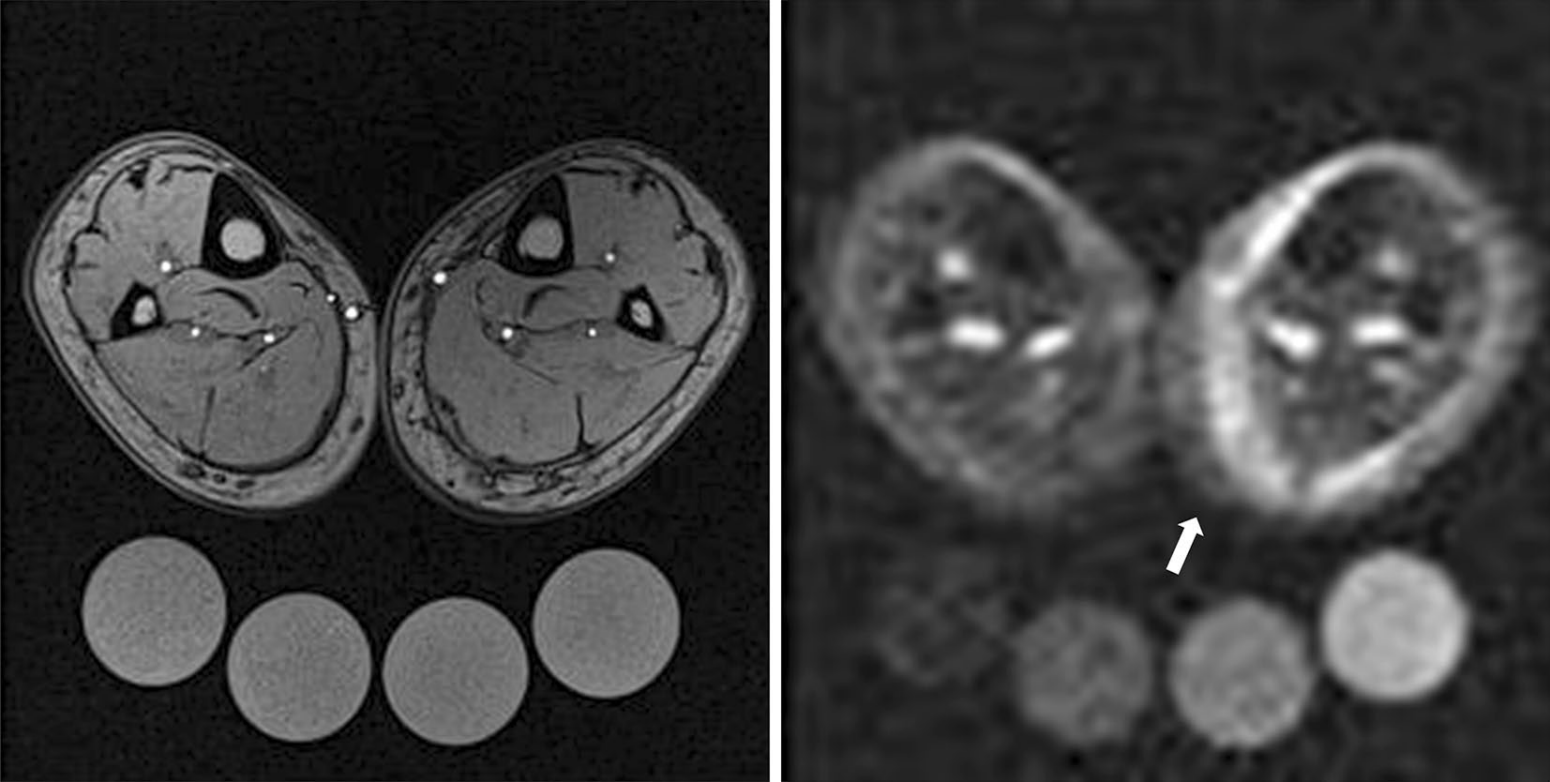
^1^H-MR imaging [T1-weighted fast-low-angle-shot (FLASH)-sequence, *left*] and ^23^Na-MR imaging (*right*) of both lower legs immediately after injury. The *left leg* shows a *half*-*moon shaped*, hyperintense Na^+^ rich area on the medial side (*arrow*). Highest muscle Na^+^ signal could be found in the region of the left medial gastrocnemius muscle (there was a 2.4-fold increase in Na^+^ concentration compared to the corresponding soft tissue of the contralateral non-affected leg, 43.5 vs. 18.0 mmol/l). The calibration tubes below the lower legs contain 10, 20, 30 and 40 mmol/l NaCl. Gray-scale measurements of the tubes served as calibration standards for ^23^Na-MRI by relating intensity to a concentration in a linear trend analysis

In the conventional T1 weighted ^1^H image (Fig. 1, left), all anatomic compartments can be seen in detail, while all Na^+^ calibration tubes appear at a similar intensity. The affected left lower leg presented a discrete swelling of the subcutaneous region in comparison to the contralateral leg. Muscle tissue itself seemed not to be affected and there is no sign of hemorrhagic bleeding. The concomitant ^23^Na-MRI showed a strong hyperintense signal in the affected leg region (Fig. 1, right), indicating increased local Na^+^ concentration. There was a 2.4-fold increase in Na^+^ concentration in the half-moon shaped region containing the medial left *triceps surae* muscle and adjacent tissue, compared to the corresponding soft tissue of the contralateral non-affected leg (Na^+^ 43.5 vs. 18.0 mmol/l). The hyperintense region was separated from the neighboring regions by manual outlining by a radiologist. The same region was used in the follow-up signal measurements.

**Fig. 2:**
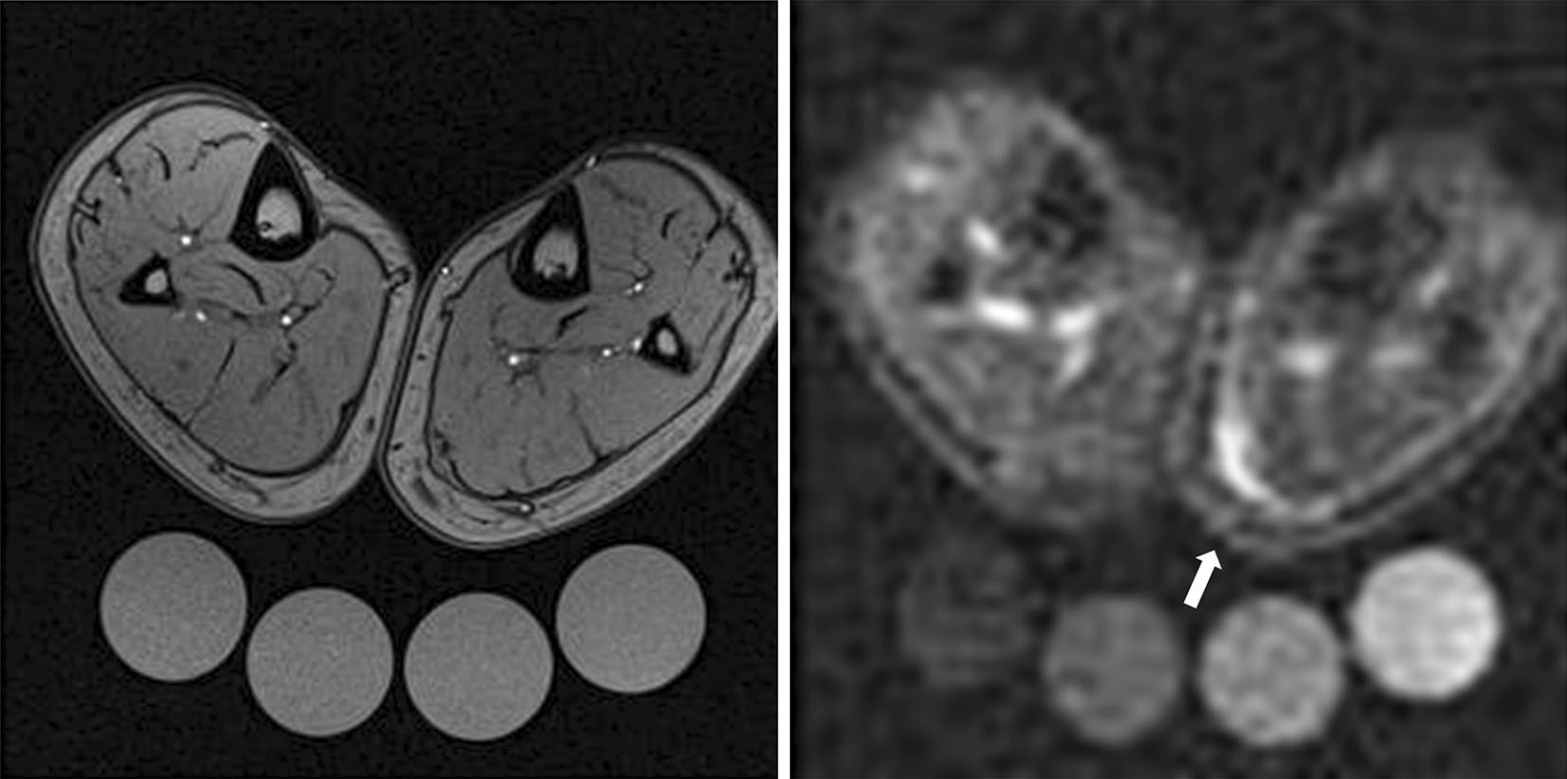
^1^H-MR imaging [T1-weighted fast-low-angle-shot (FLASH)-sequence, *left*] and ^23^Na-MR imaging (*right*) of both lower legs 2 weeks after injury. The area of hyperintense Na^+^ rich tissue was reduced, but could still be clearly visualized at the level of the left medial gastrocnemius muscle (*arrow*, Na^+^ concentration compared to the corresponding soft tissue of the contralateral non-affected leg: 37.5 vs. 18.5 mmol/l). Muscle function of the leg was completely restored by this point

**Fig. 3:**
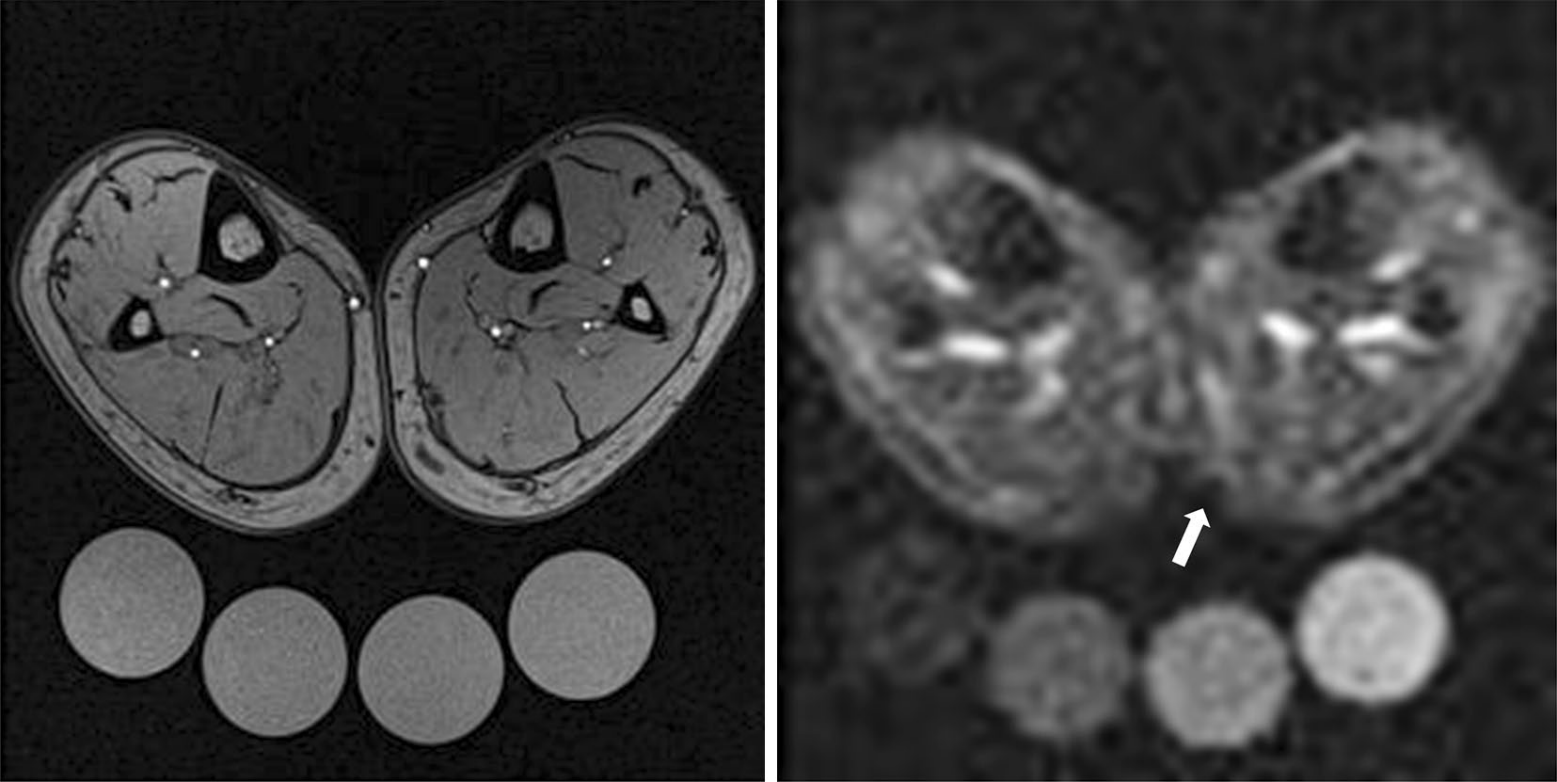
^1^H-MR imaging [T1-weighted fast-low-angle-shot (FLASH)-sequence, *left*] and ^23^Na-MR imaging (*right*) of both lower legs 2 month after injury showed only a marginal Na^+^ rich region in the medial aspect of the left gastrocnemius muscle (*arrow*, Na^+^ concentration compared to the corresponding soft tissue of the contralateral non-affected leg: 21.5 vs. 18.5 mmol/l)

Two weeks later, the patient had recovered completely. We performed follow-up imaging (Fig. 2). The Na^+^ concentration of the medial *gastrocnemius* decreased but was still elevated (Na^+^ 37.5 vs. 18.5 mmol/l, Fig. 2, right). We presume some degree of subclinical injury remained. Two months after injury, ^23^Na-MR imaging showed a barely detectable Na^+^ rich region in the medial *gastrocnemius* (Na^+^ 21.5 vs. 18.5 mmol/l, Fig. 3, right).

Based on our measurements, we assume that muscle fibers of the left medial *gastrocnemius* were torn, as the highest Na^+^ concentration was found in this region. Moreover, Na^+^ elevation in this muscle might not only be based on edema, but also on disrupted membrane potential with subsequent intracellular Na^+^ influx. ^23^Na-MRI examinations with protocols that can differentiate between intra and extracellular Na^+^ could be developed to address this notion further. Other investigators have used ^23^Na-MR imaging to study Na^+^ accumulation in patients with Duchenne’s muscular dystrophy and even to test the value of eplerenone treatment for this condition (Lehmann-Horn et al. 2012; Weber et al. 2011).

Our patient happened to have an injury that could easily be investigated with the coil we developed for our studies on Na^+^ metabolism. However, surface coils and coils of other configurations could be developed to study the upper leg, shoulder, back and other body components. Such tools could have utility in quantitatively assessing sports-related injuries and also responses to treatments. They could help in establishing *restitutio ad integrum* and thereby assist physicians in determining when players can safely return to the field.

## Conclusions

Our findings suggest that ^23^Na-MRI could have utility in quantitatively detecting subtle muscular injury and might indicate when complete healing has occurred. Furthermore, ^23^Na-MRI suggests the presence of substantial injury-related muscle electrolyte shifts that warrant more detailed investigation.

## Abbreviation

MRI: magnetic resonance imaging

## References

[CR1] Dahlmann A, Dörfelt K, Eicher F, Linz P, Kopp C, Mössinger I, Horn S, Büschges-Seraphin B, Wabel P, Hammon M, Cavallaro A, Eckardt KU, Kotanko P, Levin NW, Johannes B, Uder M, Luft FC, Müller DN, Titze JM (2015). Magnetic resonance-determined sodium removal from tissue stores in hemodialysis patients. Kidney Int.

[CR2] Hammon M, Grossmann S, Linz P, Kopp C, Dahlmann A, Garlichs C, Janka R, Cavallaro A, Luft FC, Uder M, Titze J (2015). 23Na magnetic resonance imaging in the lower leg of acute heart failure patients. PLoS ONE.

[CR3] Hammon M, Grossmann S, Linz P, Kopp C, Dahlmann A, Janka J, Cavallaro A, Uder M, Titze J (2015). 3 Tesla 23Na-Magnetic resonance imaging during aerobic and anaerobic exercise. Acad Radiol.

[CR4] Kopp C, Linz P, Hammon M, Schofl C, Grauer M, Eckardt KU, Cavallaro A, Uder M, Luft FC, Titze J (2012). Seeing the sodium in a patient with hypernatremia. Kidney Int.

[CR5] Kopp C, Linz P, Wachsmuth L, Dahlmann A, Horbach T, Schofl C, Renz W, Santoro D, Niendorf T, Muller DN, Neininger M, Cavallaro A, Eckardt KU, Schmieder RE, Luft FC, Uder M, Titze J (2012). (23)Na magnetic resonance imaging of tissue sodium. Hypertension.

[CR6] Kopp C, Linz P, Dahlmann A, Hammon M, Jantsch J, Muller DN, Schmieder RE, Cavallaro A, Eckardt KU, Uder M, Luft FC, Titze J (2013). ^23^Na magnetic resonance imaging-determined tissue sodium in healthy subjects and hypertensive patients. Hypertension.

[CR7] Lehmann-Horn F, Weber MA, Nagel AM, Meinck HM, Breitenbach S, Scharrer J, Jurkat-Rott K (2012). Rationale for treating oedema in Duchenne muscular dystrophy with eplerenone. Acta Myol.

[CR8] Weber MA, Nagel AM, Jurkat-Rott K, Lehmann-Horn F (2011). Sodium (23Na) MRI detects elevated muscular sodium concentration in Duchenne muscular dystrophy. Neurology.

